# Clinical Impact of Sugammadex in the Reversal of Neuromuscular Blockade

**DOI:** 10.7759/cureus.15413

**Published:** 2021-06-03

**Authors:** Joshua A Goodner, Eric J Likar, Abigail L Hoff, Jeffrey M Quedado, Arpan Kohli, Pavithra Ellison

**Affiliations:** 1 Pharmacology and Therapeutics, West Virginia University School of Medicine, Morgantown, USA; 2 Anesthesiology, West Virginia University School of Medicine, Morgantown, USA

**Keywords:** neuromuscular blockade (nmb), post-anesthesia discharge, reintubation, sugammadex, paralytic reversal, neostigmine

## Abstract

Background

A neuromuscular blockade (NMB) is used in general anesthesia to facilitate endotracheal intubation and muscle relaxation during procedural and surgical interventions. Rapid and complete reversal of the NMB allows for patient recovery to the preoperative baseline with ventilation and motor function, along with the complete return of gastroesophageal motility, thereby expediting recovery and preventing microaspiration in the postoperative period. Sugammadex is a modified gamma cyclodextrin that complexes with steroidal neuromuscular blocking agents (specifically, rocuronium and vecuronium), leading to a molecular gradient and removal of the agents from the neuromuscular junction. Sugammadex has been shown to have a more rapid reversal of neuromuscular blockade compared to neostigmine. The purpose of this study was to evaluate if perioperative efficiency was increased when sugammadex was used for paralytic reversal compared to the traditional regimen of neostigmine and glycopyrrolate.

Methods

A retrospective cohort study of patients admitted for surgical intervention in June 2019 was conducted. Two groups were compared: those who received sugammadex for reversal and those who received neostigmine, plus glycopyrrolate. The primary outcome was time to extubation from the administration of the reversal agent.

Results

Two hundred seventy-one surgical cases were evaluated. Average doses of sugammadex for those with profound neuromuscular blockade as indicated by a train of four (TOF) of 0 - 2 was 2.47 (0.9) mg/kg for sugammadex and 0.042 (0.01) mg/kg for neostigmine, plus glycopyrrolate. Seventeen patients in the sugammadex group experienced bradycardia after reversal compared to 22 in the neostigmine, plus glycopyrrolate, group (p = 0.73*).* Reintubation was required for three patients in the neostigmine, plus glycopyrrolate, group and no patients in the sugammadex group.

The mean time to extubation from the procedure end comparing reversal with sugammadex and neostigmine, plus glycopyrrolate, was 12.5 (7.6) minutes versus 13.7 (8.8) minutes (p* *= 0.44), respectively. Comparison of reversal with sugammadex versus neostigmine, plus glycopyrrolate, and time spent in the post-anesthesia care unit was 83.6 (48.6) minutes versus 81.7 (46.6) (p = 0.73), respectively.

Conclusions

In this retrospective cohort study, we observed a deviation in the recommended sugammadex dosage and increased reintubation rates but no difference in time to extubation or Post-Anesthesia Care Unit (PACU) length of stay times when patients received sugammadex compared to neostigmine, plus glycopyrrolate, for neuromuscular blockade reversal. Understanding the PACU flow and culture, education of providers about dosages, along with completion of prospective studies, to correlate acceleromyograph values to reversal and postoperative ventilatory and deglutary function can help assess the true clinical value of sugammadex.

## Introduction

Sugammadex is a reversal agent used to counteract steroidal paralytic medications. As a modified gamma-cyclodextrin, it forms a complex via encapsulation with rocuronium and vecuronium. This subsequently decreases the amount of the neuromuscular blocking agent that can bind to nicotinic receptors in the neuromuscular junction. Due to its unique mechanism of action, it has an onset of two minutes and a half-life of two hours [1]. Comparatively, another medication commonly used as a paralytic reversal agent in clinical practice is neostigmine. As an acetylcholinesterase inhibitor, neostigmine works to suppress the enzymatic breakdown of acetylcholine. This allows the molecule to accumulate and displace vecuronium or rocuronium on nicotinic receptors. Due to the anticholinergic side effects of neostigmine, this medication is commonly given with glycopyrrolate. However, in a variety of clinical scenarios, neostigmine alone may not be fully effective. If the neuromuscular blocking agent concentration is profoundly high, the increase in acetylcholine will be insufficient to displace enough neuromuscular blocking agents to reverse the blockade [2]. The other most common untoward side effects of neostigmine, apart from bradycardia, are hypotension, nausea, vomiting, and headache [1].

Train of four (TOF) monitoring is used to determine the timing that a reversal agent should be administered and if subsequent doses are needed. A TOF twitch ratio of 90% or greater is considered clinically significant for reversal and should be achieved at the end of surgery prior to tracheal extubation [3]. Patients who undergo premature extubation in the immediate postoperative period are at a higher risk of airway obstruction, pulmonary complications, and other significant morbidities [3-4]. In a randomized, controlled trial of the reversal of rocuronium-induced neuromuscular blockade with sugammadex compared with neostigmine, the time to recovery of a 0.9 TOF ratio was significantly shorter, 1.5 minutes versus 18.6 minutes, respectively (P < 0.0001) [5]. Additionally, the predictability of the responses was greater with sugammadex. Ninety-eight percent of patients (n = 49) recovered to a TOF ratio of 0.9 within five minutes, as compared to 11% of neostigmine patients (n = 49) [4-5]. Decreased Post-Anesthesia Care Unit (PACU) time decreases costs for both the patient and hospital, but it is unknown if this is truly realized in clinical practice. We completed a retrospective study to evaluate whether there is a clinical advantage to the use of sugammadex over neostigmine, plus glycopyrrolate.

## Materials and methods

Study design

This was a retrospective chart review of patients admitted to our institution for surgical intervention in June 2019. The Institutional Review Board (IRB) at West Virginia University School of Medicine waived approval for this study (#1905564980). All patients greater than 18 years of age were identified via a medication order search for either sugammadex or neostigmine and glycopyrrolate in the electronic medical record (EMR). Patients were randomly selected from the resulting list for review. The selection of patients was via random number generation whereby the patients were randomly assigned a value and the top 150 patients from each group were selected. In addition to baseline demographics, the following data points were collected for each patient: paralytic agent, dose, and administration time; paralytic reversal agent selection, dose, and administration time; operative case end time, time of extubation, time patient exited the OR, train of four (TOF) monitoring during surgery, time the patient met PACU discharge criteria, operating room location, surgery type, medication charge, the occurrence of reintubation, and occurrence of bradycardia, defined as a heart rate less than 60 beats per minute.

Study population

One-hundred and fifty patients were selected from the ambulatory operating room (OR) suites and 150 from the inpatient OR suites for a total of 300 patients. Patients were included if they were 18 years of age or older, received a single dose of sugammadex or neostigmine and glycopyrrolate, and went to the PACU after procedure conclusion. Patients were excluded if they were discharged directly from the OR suite without going to the PACU, had a documented allergy to sugammadex, neostigmine, or glycopyrrolate, or if they remained intubated after procedure completion.

Outcome measures

The primary outcome of this study was a mean calculated absolute difference between documented procedure end time and documented time of extubation. Secondary outcomes assessed included trends in PACU recovery time between agents, trends in drug selection based on TOF monitoring, and trends in the dosing of paralytics and reversal agents. Adverse event rates were characterized for each treatment regarding bradycardia, defined as a heart rate less than 60 beats per minute, and the occurrence of reintubation.

Statistical analysis

Baseline characteristics were compared descriptively in line with Consolidated Standards of Reporting Trials (CONSORT). Descriptive statistics are presented as mean (standard deviation (SD)), minimum-maximum or median (interquartile range (IQR)), and Valid N if data was missing. Categorial data is presented as N (%) and the Valid N if data is missing. The primary outcome (the mean time to extubation from the procedure end) was assessed via a Wilcoxon non-parametric test. For secondary outcomes, the Wilcoxon test was used to assess mean PACU time and paralytic and reversal agent doses. Drug selection based on TOF monitoring was assessed using Chi-square analysis. The presence of adverse events, bradycardia, and reintubation was assessed using Chi-square analysis. A p-value of < 0.05 was considered to indicate statistical significance.

## Results

Eight-hundred and fifteen cases were identified; 150 cases completed in the inpatient operating room suites and 150 cases completed in the ambulatory operating room suites were selected for review. Of the 300 cases, 271 were included in the analysis (134 cases from the inpatient operating room suites and 137 cases from the outpatient operating room suites); 29 cases met exclusion criteria and were excluded from evaluation (Table 1).

**Table 1:** Characteristics of Patients Who Received Sugammadex or Neostigmine SD: standard deviation

Characteristics	Overall (n = 271)	Sugammadex (n = 117)	Neostigmine (n = 154)	P-Value
Average age in years ± SD	55.3 ± 17.0	55.8 ± 16.2	54.8 ± 17.5	0.770
Female sex n (%)	152 (56)	69 (59)	83 (54)	0.404
Inpatient n (%)	132 (49)	60 (51)	72 (47)	0.492
Outpatient n (%)	139 (51)	57 (49)	82 (53)	0.492

Primary outcome

The mean time to extubation for the cases in which sugammadex was used was 12.5 (7.6) minutes compared to 13.7 (8.8) minutes for cases where neostigmine and glycopyrrolate were used (p = 0.44). In a subgroup analysis of patients with deep or profound neuromuscular blockade, as indicated by a TOF of 0 - 2, the mean time to extubation was 13.6 (10) compared to 13.2 (11) for patients receiving sugammadex and neostigmine, plus glycopyrrolate, respectively (p = 0.55).

Secondary outcomes

The average PACU time for patients receiving sugammadex was 83.6 (48.6) minutes compared to 81.7 (46.6) in those who received neostigmine and glycopyrrolate (p = 0.73). Similar trends were observed in a subgroup analysis based on whether the procedure occurred in the inpatient operating room suites or the outpatient operating room suites. The average doses of the reversal agent used are shown in Table 2.

**Table 2:** Dosages Utilized for Reversal of Neuromuscular Blockade with Sugammadex or Neostigmine SD: standard deviation

Dosage (mg/kg ± SD)	Sugammadex (n = 117)	Neostigmine (n = 154)
Average	2.05 ± 0.92	0.042 ± 0.011
Inpatient	2.29 ± 0.91	0.044 ± 0.011
Outpatient	2.67 ± 0.89	0.040 ± 0.011
Profound block	2.47 ± 0.9	0.042 ± 0.01

The average doses of sugammadex for those with profound neuromuscular blockade as indicated by a TOF of 0 - 2 was 2.47 (0.9) mg/kg for sugammadex and 0.042 (0.01) mg/kg for neostigmine. Seventeen patients in the sugammadex group experienced bradycardia after reversal compared to 22 in the neostigmine, plus glycopyrrolate, group (p = 0.73). Reintubation was required for three patients in the neostigmine, plus glycopyrrolate, group and no patients in the sugammadex group. Seventy-six patients included in the analysis did not have a TOF measurement recorded prior to reversal.

## Discussion

In this retrospective study, we did not elicit a time-saving effect of utilizing sugammadex versus neostigmine. There was a higher reintubation rate in the neostigmine group (3 versus 0). One potential explanation for this observation is the observed dosing of sugammadex was lower than required, especially for patients with profound neuromuscular blockade [5]. Furthermore, patients in this study were noted to have either minimal blockade (by TOF monitoring) or unreported levels of blockade which may be due to underreporting or misreporting of TOF values. Sugammadex has been previously shown to allow for rapid reversal to a TOF of 4/4, but to our knowledge, its efficacy in those with minimal blockade has not been evaluated in the literature. As noted previously, doses of reversal agents (specifically, sugammadex) were found to be lower in our study than those reported in previously published studies. This may have been a function of TOF monitoring prior to reversal, with providers selecting lower doses for patients with minimal levels of neuromuscular blockade.

Rates of bradycardia seen in this study were higher than those reports in the literature for sugammadex [1]. This is possibly due to the retrospective nature of our study, as well as its pragmatic approach which did not limit other factors, including medications that may have contributed to bradycardia. Finally, rates of reintubation observed in our cohort were low overall with no patients receiving sugammadex requiring reintubation and three patients in the neostigmine group requiring reintubation.

Our study reflects the real-world issues related to TOF value reporting, knowledge on accurate dosing, and PACU culture which can affect efficiency and flow.

Our study has several limitations. Chiefly among these is its retrospective nature and small sample size. Patient population heterogeneity could also be viewed as a limitation as our cohort population included both ambulatory and inpatient status procedures. This could have confounded our results due to the more rapid PACU turnaround time preferred in the outpatient setting compared to the inpatient setting where patients may be boarded in the PACU prior to returning to the floor. Another limitation of this study is the observed dosing of reversal agents. As noted previously, in other studies, the sugammadex dosing for patients found to have profound blockade was 4 mg/kg. This is significantly higher than the doses seen in comparable patients in our study where an average dose of 2.5 mg/kg was used. Finally, the broad inclusion criteria used in our study may have prevented us from detecting a difference in those who may have most benefited from sugammadex administration, including those with deep or profound neuromuscular blockade.

## Conclusions

In this retrospective study, the reversal of the NMB with sugammadex was not associated with decreased time to extubation or decreased length of stay in the PACU. Although the best strategy and optimizing patients may help, the overall improvement in the flow of patients and better education of postoperative teams will be necessary to overcome the dogma of PACU discharge times that are correlated to qualitative data and assessments, such as meaningful utilization of the Alderette criteria for discharge. Future prospective studies should focus on the pharmacoeconomic impact analysis based on recovery times, as our study was unable to detect a difference concerning this variable. Furthermore, additional studies are necessary to better understand the implications of NMB reversal with sugammadex regarding patient outcomes and the true value created.
